# Exploring the influence of long COVID upon self-identity: a systematic literature review

**DOI:** 10.1186/s13690-026-02017-8

**Published:** 2026-08-11

**Authors:** Helena J. Holmes-McCoid, Fiona E. Lecky

**Affiliations:** 1 School of Medicine and Population Health, Faculty of Health, University of Sheffield, Sheffield, UK; 2 Department of Psychological Services, Mid Yorkshire Teaching NHS Trust, Wakefield, UK; 3 Northern Care Alliance NHS Foundation Trust, Salford, UK

**Keywords:** Narrative synthesis, Long COVID, Self-identity, Self-concept, Illness-identity

## Abstract

**Background:**

Long COVID (LC) is understood to be a multisystemic illness involving symptoms of an enduring nature. As underlying pathophysiological mechanisms of LC become better understood, attention has turned towards the psychological aspects of LC lived experience. Comparable conditions, such as myalgic encephalomyelitis/chronic fatigue syndrome (ME/CFS), are known to significantly impact self-identity. However, no prior review has explored this topic in the LC population. This review sought to offer an original and exploratory narrative of LC patients’ experiences connected to self-identity.

**Methods:**

A systematic literature review using a Narrative Synthesis approach. Twenty-one qualitative studies (including 613 participants), published between January 2021–March 2025, exploring the influence of LC upon perceptions of self-identity were reviewed. Narrative Synthesis (NS) and PRISMA guidelines were followed.

**Results:**

Four main themes emerged: “Grief and Loss of the Familiar Self”, “Reflections of Self, Mirrored by Others”, “Threats to Identity”, and “Repairing Fractured Identity”.

**Conclusions:**

For many, LC ruptures self-narratives that contribute to a stable and continuous sense of self across time. This can bring forth a strong grieving response to perceived losses and the unfamiliarity of self. Despite this, narratives of post-traumatic growth (PTG) were realised. Current guidance lacks direction for psychological interventions targeting LC-related distress. Acknowledgement of identity-based challenges must also be reflected in future guidance, with clear recommendations for clinical practice. Qualitative longitudinal exploration of LC’s influence upon self-identity should be prioritised. Similarly, the acceptability and feasibility of interventions targeting identity-transformation work require further investigation.

**Trial registration:**

PROSPERO ID: CRD420251008410.

**Supplementary Information:**

The online version contains supplementary material available at 10.1186/s13690-026-02017-8.

**Table:** 

Text box 1. Contributions to the literature
• This study focuses on self-identity in relation to long COVID – a sparsely studied topic in an underserved population. Valuable insights into the psychological influence of long COVID are provided, highlighting notable changes to self-identity for many individuals adjusting to this condition
• Demonstrates the importance of prioritising identity-focused therapy with long COVID patients
• Provides data cable of informing clinical practice and best practice guidelines for psychological practitioners working with long COVID patients
• Offers potential direction for future research and clinical practice, identifying promising therapeutic modalities

## Background

Post-COVID-19 Syndrome, commonly termed Long COVID (LC), is a condition involving multisystemic symptoms lingering beyond twelve weeks post-COVID-19 infection [1]. For example, many individuals experiencing LC report ongoing fatigue, dyspnoea (breathlessness), sleep problems, headaches, musculoskeletal pain, ‘brain fog’, and executive dysfunction [2, 3]. Additionally, COVID infection reportedly contributes to the onset of secondary conditions in a portion of the LC population [4]. More than 400 million people have developed LC since the 2020 pandemic commenced, with current global estimates indicating that 6-in-100 COVID infections result in LC [5, 6]. Recent research has explored lived experience of navigating this condition, highlighting negative accompanying psychological sequelae [7–9]. Such research underscores the psychosocial challenges impacting most aspects of life when navigating adjustment to LC, e.g., employment, finances, social functioning, interpersonal relationships, psychological well-being, and quality of life [10–13]. Owing to these significant personal costs, coupled with United Kingdom (UK) estimates of doubled healthcare expenditure per LC patient, it is essential to wholly understand this condition to establish effective rehabilitation and care [14, 15].

There is a dominant narrative in burgeoning literature that LC-related experiences constitute a biographical disruption, which engenders grieving for losses, a need to redefine ‘the self’, and a process of reevaluating life priorities, values, and goals [16, 17]. Often, those with LC struggle to reconcile their ‘former self’ (perception of self-identity before LC) with their ‘current self’ (present-day understandings of self-identity) [18–20]. Moreover, added uncertainty surrounding LC’s non-linear illness trajectory may further obstruct a clear mental representation of the ‘future self’ [18, 21]. In established literature, this experiential phenomenon is broadly described as changes to “identity”, “self-identity”, “sense of self”, “selfhood”, or “personal identity” – these terms are used interchangeably hereon [22, 23]. The closely related umbrella term “self-concept”, the entirety of an organised, complex, and modifiable system of evaluative perceptions, beliefs, and learned attitudes that individuals form about themselves, is equally relevant and referenced where appropriate [24].

Despite recognition of LC’s influence upon self-identity, an in-depth exploration of existing literature delineating this topic remains absent. Understanding all psychological aspects of adjustment to LC is imperative to guide appropriate provision of psychological care: especially in the current context of limited professional practice guidelines [8, 25–27]. However, self-identity has been explored to varying degrees within existing LC-related literature. Most studies prioritise a broader focus but still touch upon this subject. Few articles solely address self-identity in LC [17, 18]. Papers discuss a lost sense of self generated by changes to physical ability and living in a body that no longer feels, and in some instances looks, familiar [7, 17, 28–30]. Other articles highlight altered identity through a disconnect with specific qualities of perceived personality, e.g., no longer viewing oneself to be “fun” [20].

Published LC research further explores self-identity transformation in the context of changes to, or loss of, established personal roles, i.e., role as a parent, worker, spouse, caregiver, friend etc [31–33]. Ruptures, reduced contact, and losses within relationships, framed as a consequence of revisions to these personal roles, have also been described [34]. Loss of the expectation of assuming a future role due to LC, for example the role of becoming a parent, may further disrupt sense of self [17, 34]. Role changes also relate to withdrawal of employment and adjustment to working roles – critically pertinent to those with a strongly held professional identity [21, 35, 36].

The challenge of integrating new aspects of the self into self-identity, e.g., viewing oneself as a person living with a chronic condition or as a recipient of care, has further been established [7, 37, 38]. The issue of separating illness from self-identity is well acknowledged [17]. Yet, simultaneously, identity reconstruction including a narrative of the self with LC can be taxing to initiate, partly due to societal stigma [16, 39]. LC patients often describe invalidating responses to their experience of the condition, from suspicion and dubiousness regarding the diagnosis and its validity, to vocal denial of its existence [40]. Alarmingly, presence of this attitude has been described within healthcare systems [31]. On a broader scale, the lack of recognition of LC from governments and media institutes further intensifies this societal stigma [35].

For some, the numerous strains outlined above decrease motivation to engage with previously enjoyed activities of daily living (AoDL), behaviours that contribute to sense of identity, even when LC symptoms are not a wholly obstructing barrier [23, 41, 42]. Fang et al [18] suggest that a ‘disrupted chronology’, i.e., lack of understanding and familiarity of self in the present, lack of cohesion with ‘past self’, and changing expectations of the ‘future self’, perpetuates issues of self-identity in those with LC. Thus, resulting in ‘temporal stuckness’, or a quality of life feeling ‘paused’ [33].

Finally, research touching upon the topics of LC and self-identity considers the broad spectrum of LC-related experience – including positive psychological transformation [17, 23, 33, 43]. It is understood that positive psychological change can occur following traumatic, significantly distressing, and often highly challenging life circumstances – termed post-traumatic growth (PTG) – which may emerge following a perceived threat to self-identity, e.g., a pivotal biographical disturbance (LC) that functions as a catalyst for sensemaking and expansion of self-narratives [44]. Crucially, psychological practitioners can support reflection on these processes through therapeutic input [45–47]. However, a full appreciation of how alterations to self-identity are experienced through LC illness is firstly required. Accordingly, the current review intended to address this research gap.

### Current review: objectives

This review had two core aims: a) to offer a previously unexplored narrative of LC patients’ experiences connected to self-identity, and b) to yield data capable of informing and improving LC-related care – in particular, the provision of psychological support.

The review question identified was as follows: what influence does LC have upon perceptions of self-identity?

## Methods

### Search strategy

Papers describing lived experience pertaining to LC’s influence upon self-identity were sought. Several electronic databases (MEDLINE, CINAHL, APA PsycArticles, APA PsycInfo and the Core Collection of the Web of Science) were searched to secure qualitative research, published between January 2021 and March 2025, focused on first-hand reports of the topic of interest. This search strategy encompassed psychological, medical, and general databases to ensure a broad search scope, enhancing the probability of relevant article detection.

Search terms were comprehensive and varied, minimising the risk of omitting applicable literature (see “Supplementary Materials”, SM). Asterisk truncations, following root words, ensured inclusion of connected word variations across search results. Quotation marks captured specific terms and phrases. Boolean operators (“OR”, “AND”) retrieved suitable articles by broadly searching factors connected to the research question.

Initially, the search process was completed independently by the first author (HH-M). An identical database search was performed by Mid Yorkshire Teaching NHS Trust’s library searching service thereafter. Both sets of search data were scrutinised and contrasted. However, no new articles were detected through comparison of search results.

### Study inclusion and exclusion criteria

**Fig. 1:**
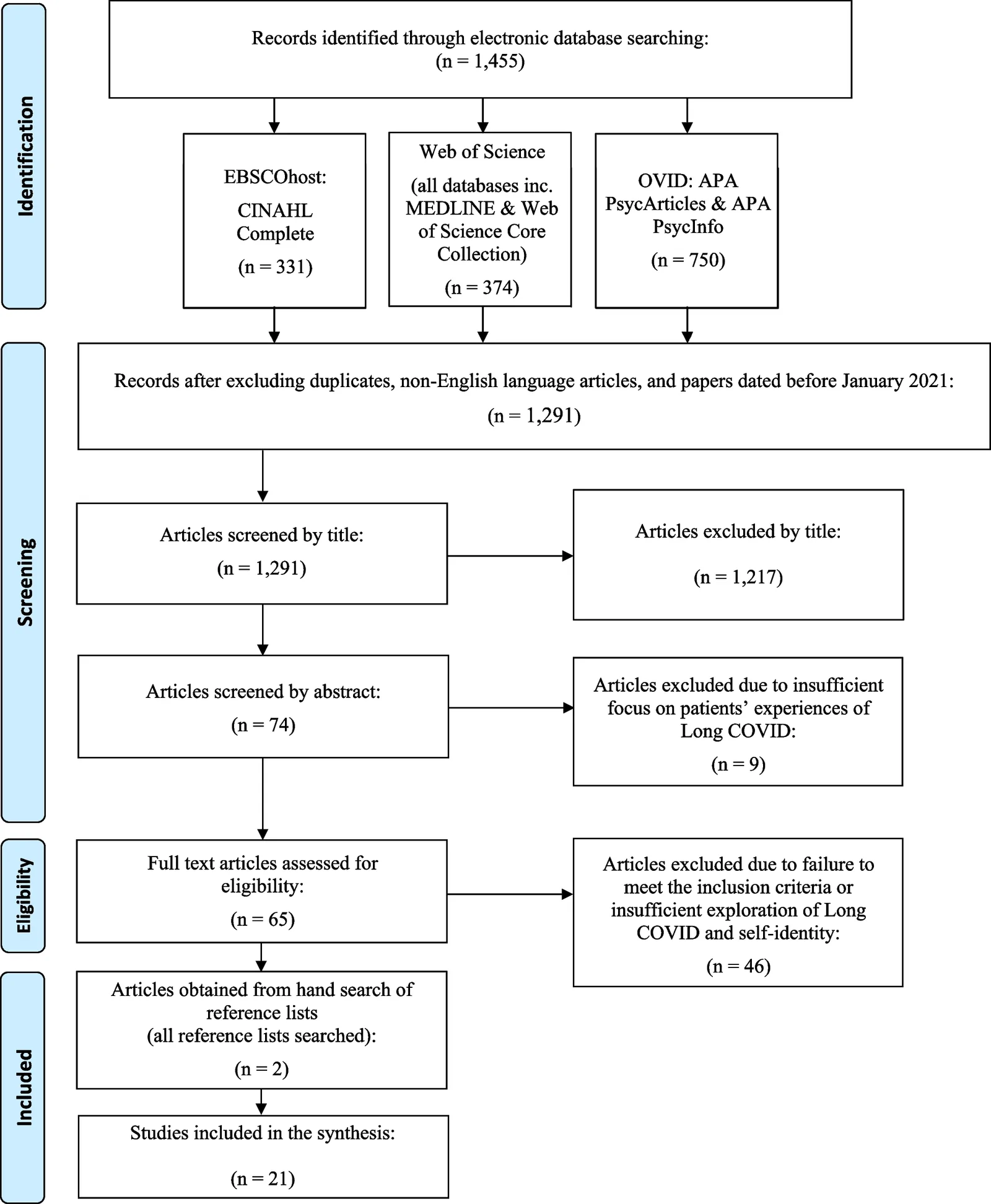
Article selection process. Note. Figure adapted from “PRISMA 2009 Flow Diagram” [48].

The selection process was guided by the Preferred Reporting Items for Systematic Reviews and Meta-Analyses (PRISMA) statement [48] illustrated in Fig. 1 and completed by the first author (HH-M). Papers included after duplicate removal and screening (title, abstract or full text) were evaluated against the exclusion and inclusion criteria (see Tables 1 and 2). The reference lists, of all qualifying papers, were perused to obtain undiscovered articles.

**Table 1:** Inclusion criteria and rationales

Inclusion Criteria	Rationale
Peer reviewed	Peer reviewed articles are thoroughly evaluated by the scientific community, yielding high-quality content [49].
Published between January 2021–March 2025	To cover the timeframe in which literature emerged relating to LC (the cut-off date allowed synthesis against a feasible project timeframe).
Articles focused on, or including underlying themes relating to, first-hand perceptions of self-identity connected to LC lived experience	To meet the core aims of this review.
English language papers	Due to limited resources, translation services were inaccessible. The first author is English speaking, only. Hence, solely articles written in English, or already translated to English, were analysed and synthesised.
Relevant first-hand experiences of individuals experiencing LC	Allowing for the nuance, richness and complexity of lived experience to be captured within the synthesis.
Qualitative methodology	Qualitative methodologies allow for depth of lived experience to be examined – particularly apt when synthesising novel data on an under-researched topic.
Papers with data derived from direct interaction between participants and the researcher(s)	Reliability and validity of findings may be enhanced by synthesising data ‘closest’ to participants’ accounts (and therefore experiences). Thus, excluding literature reviews. Data-rich qualitative reports, derived from survey data, may be included.

**Table 2:** Exclusion criteria and rationales

Exclusion Criteria	Rationale
Quantitative and mixed-method approaches	To enable the review question to be appropriately answered. Quantitative methodologies are not designed to capture the detail or nuance of lived experience. Arguably, mixed-method approaches do so to a lesser extent than qualitative methodologies [50].
Articles with a narrow focus on a specific element of the LC experience that lacks relevance to the topic of interest	To meet the aims of the current review.
Systematic literature reviews	Findings of systematic literature reviews represent second-hand interpretations of first-hand original sources. The current review aimed to reflect the original data sources as much as possible.
Non-English language papers	Due to limited resources, translation services were inaccessible. Therefore, non-English language papers were excluded.

### Quality assessment

Utilisation of the National Institute for Health and Care Excellence (NICE) Quality Assessment Checklist [51] enabled a standardised and systematic assessment of articles’ methodological quality (see SM, Appendices A and B, for rating categorisations and an example checklist). This checklist was selected owing to its depth of assessment, and simplistic rating system. An overall quality rating was determined for each article, derived from a rigorous review of 14 domains. The second author (FL) separately evaluated the quality of three articles, functioning as a reliability check of the first reviewer’s findings. Comparison between ratings was discussed, although no differences in scoring were observed. Given the unanimity between raters, remaining articles were assumed to be reliably rated by the first reviewer (HH-M). See Table 3 for articles’ quality ratings.

**Table 3:** Included studies: overview

No	Author, (Year), ‘Title’	Location	Method	Participant Demographics: **Sample Size** = Gender Mean Age (Age Range)
1	Burton et al., (2022) [7] ‘Factors Shaping the Mental Health and Well-Being of People Experiencing Persistent COVID-19 Symptoms or ‘Long COVID’: Qualitative Study’	UK	Reflexive TA – Semi-structured interviews	**21** = 14 Females, 7 others (no description) 47 Years (26–70)
2	Collier & Garip (2023) [28] ‘The Hidden Pandemic: A Qualitative Study on How Middle-Aged Women Make Sense of Managing Their Long COVID Symptoms’	UK	IPA – Semi-structured interviews	**9** = 9 Females 50.60 Years (42–59)
3	Fang et al., (2024) [43] ‘“I Am Just a Shadow of Who I Used To Be”—Exploring Existential Loss of Identity Among People Living with Chronic Conditions of Long COVID’	UK	Narrative Analysis – Narrative, semi-structured interviews	**80** = 56 Females, 24 males Mean age not reported (18–75)
4	Humphreys et al., (2021) [41] ‘Long COVID and the Role of Physical Activity: A Qualitative Study’	UK & USA	Inductive TA – Semi-structured interviews	**18** = 9 Females, 9 males Mean age not reported (18–74)
5	Hunt et al., (2024) [42] ‘Young Adults’ Experiences of Biographical Retrogression Whilst Living with Long COVID’	UK	TA – Semi-structured interviews	**15** = 10 Females, 5 males 25.2 Years (20–32)
6	Goodridge et al., (2023) [31] ‘“We’re Drowning and We’re Alone”: A Qualitative Study of the Lived Experience of People Experiencing Persistent Post-COVID-19 Symptoms’	Canada	Reflexive TA – Semi-structured interview guide used in focus groups	**41** = 28 Females, 13 males 47.9 (29–83)
7	Kalfas et al., (2024) [37] ‘Exploring the Experiences of Living with the Post-COVID Syndrome: A Qualitative Study’	UK	Reflexive TA – Semi-structured interviews	**19** = 13 Females, 6 males 50 Years (30–78)
8	Kennelly et al., (2023) [34] ‘The Lived Experience of Long COVID: A Qualitative Study of Mental Health, Quality of Life, and Coping’	Canada	TA – Semi-structured interviews in focus groups	**47** = 28 Females, 18 males, 1 non-binary gender Mean age not reported (18–55 >)
9	Leggat et al., (2024) [23] ‘An Exploration of the Experiences and Self-Generated Strategies Used When Navigating Everyday Life with Long Covid’	UK	Reflexive TA – Semi-structured, narrative interviews	**18** = 12 Females, 6 males Mean age & range not reported
10	Lieberworth & Niemeijer (2024) [21] ‘Lost and Changed Meaning in Life of People with Long Covid: A Qualitative Study’	Netherlands	Constructivist Grounded Theory – Semi-structured interviews	**10** = 6 Females, 4 males 30.0 Years (20–56)
11	McAlearney et al., (2024) [20] ‘A Journey Through Grief: Experiences of Loss Among Patients with Long COVID’	USA	TA – Semi-structured interviews	**21** = 16 Females, 5 males 47.6 Years (19–68)
12	Milne, Arnold & Moore (2024) [52] ‘Understanding Post-Hospitalised Patients’ Experiences of Long-COVID – The PELCO Study’	UK	TA – Semi-structured interviews	**12** = 7 Females, 5 males Mean age not reported (42–81)
13	Nielsen & Yarker (2024) [35] ‘“It’s a Rollercoaster”: The Recovery and Return to Work Experiences of Workers with Long COVID’	UK	IPA – Semi-structured interviews	**12** = 11 Females, 1 male 44.70 (30–58)
14	Petker & Ogden (2025) [53] ‘Patients’ Experiences of Living With Long Covid and Their Beliefs About the Role of Psychology in Their Condition’	UK	Inductive Reflexive TA – Semi-structured interviews	**14** = 12 Females, 2 males Mean age not reported (27–63)
15	Schmachtenberg, et al., (2023) [32] ‘How Do Long COVID Patients Perceive Their Current Life Situation and Occupational Perspective? Results of a Qualitative Interview Study in Germany’	Germany	Content Analysis – Structured interviews	**25** = 18 Females, 7 males 45 Years (21–67)
16	Skilbeck, Spanton & Paton (2023) [33] ‘Patients’ Lived Experience and Reflections on Long COVID: An Interpretive Phenomenological Analysis within An Integrated Adult Primary Care Psychology NHS Service’	UK	IPA – Semi-structured interviews	**18** = 13 Females, 5 males Mean age not reported (18–65)
17	Spence et al., (2023) [17] ‘Getting Back to Normal? Identity and Role Disruptions Among Adults with Long COVID’	USA	TA – Semi-structured, narrative interviews	**20** = 16 Females, 3 males, 1 non-binary/third gender 42 Years (20–64)
18	Taylor et al., (2021) [36] ‘“Reluctant Pioneer”: A Qualitative Study of Doctors' Experiences As Patients with Long COVID’	UK	Interpretive TA – Semi-structured, narrative interviews	**13** = 11 Females, 2 males Mean age not reported (30–50)
19	Thomas et al., (2023) [29] ‘Lived Experience of Patients with Long COVID: A Qualitative Study in the UK’	UK	Longitudinal, Observation Study via TA – Participants’ hand-written diaries (spanning 16-weeks)	**12** = 11 Females, 1 male 49 (Age range not reported)
20	Thompson et al., (2024) [54] ‘Living with Long COVID: A Longitudinal Interview Study of Individuals’ Communicative Resilience Through the “Long Haul”’	USA	TA – Semi-structured interviews	**19** = 16 Females, 2 males, 1 non-binary gender Mean age not reported (20–61)
21	Wurz et al., (2022) [30] ‘“I Feel Like My Body is Broken”: Exploring the Experiences of People living with Long COVID’	Canada/International Recruitment	Reflexive TA – Online survey with open-ended questions	**169** = 49 Females, 16 males, 3 non-binary gender, participants, 1 preferred not to answer Mean age not reported (18–79)

No	General Illness-Duration Description OR (*) Year of First COVID Infection (No. of Participants) OR ** Time Spent Living with Long COVID (Range OR No. of Participants)		Main Findings *(Subthemes)*	Quality Rating
1	** Mean of 29 weeks (range = 8–52)		1. Symptoms Causing Severe Disruption to Daily Life 2. Access to Services and Treatments 3. Uncertainty of Illness Trajectories 4. Care and Understanding from Others 5. Changes to Identity	+ +
2	Participants who, “contracted COVID from March 2020 … were treated at home and no hospital admission for COVID and had Long COVID symptoms lasting for more than 28 days” (page 272)		1. Inequality and Inconsistent Medical Treatment a. *Mistrust of Medical Professionals* b. *Validation, Support and Accessing Medical Treatment* 2. Uncertainty and Ambiguity of Managing Long COVID Symptoms a. *The Factors That Increase Long COVID Symptoms* b. *Managing Long COVID Symptoms* 3. Managing Other People’s Expectations and Perceptions of Long COVID a. *Feeling Judged and Misunderstood* b. *Positive and Meaningful Interactions* 4. The Changing Identity a. *Mourning the Old Self* b. *Hope and Gratitude*	+ +
3	“The majority … had symptoms for over 6 months with the longest … around 20 months at the time of the interviews” (page 64)		1. Disruptions to Self-Continuity 2. Barriers in Narrative Reconstruction of Identity 3. Disconnection from the Past & Perceived Future 4. Deeper Pain of Non-Being 5. Reflexively Restoring the Coherence of Self	+
4	** 3 Weeks or more (all participants)		1. The Interconnection of Physical and Psychological Symptoms 2. Lack of Clear and Consistent PA-Related Advice 3. Learning to Balance Symptoms and Activity 4. Adapting to An Altered Life	+ +
5	(*) Early 2020 (1) (*) March 2020 (5) (*) October 2020 (1) (*) December 2020 (1) (*) Late 2020 (1) (*) January 2021 (1) (*) July 2021 (2) (*) October 2021 (1) (*) December 2021 (2)		1. Moving Back—Changes and Challenges to Young Adults’ Lives and Identities Due to Long COVID 2. Disrupted Futures in the Context of a Nascent Condition with No Established Natural History	+
6	Time elapsed (in months) since acute COVID infection – mean ± SD (range): 15.8 ± 6.4 (3–27)		1. The Unique Burden of Living with Persistent Post-COVID-19 Symptoms 2. The Complex Work of Seeking Treatment 3. The Erosion of Trust in Health Care 4. The Evolving Process of Adaptation to New Realities	+
7	Data not reported		1. Symptom Dismissal 2. Lack of Information and Support 3. Life Before and After Long COVID 4. Psychological Impact 5. Acceptance	+ +
8	Time elapsed (in months) since acute infection: 3–6 (9 participants) 7–12 (15 participant) 13 + (17 participants) Missing data (6)		1. The Emotional Landscape of Long COVID a. *Anxiety and Fear of the Unknown* b. *Challenges with Emotional Regulation* c. *Feelings of Hopelessness and Depression* 2. New Limits to Daily Functioning a. *Living with Brain Fog* b. *Exhaustion As a Barrier to Completing Tasks in Everyday Life* 3. Grief and Loss of Former Identity a. *Reconciling Past and Present Identities* b. *Loss of Social Connections* 4. Long COVID-related Stigmatization a. *Trivialization of Experience with Long COVID* b. *The Burden of Blame* 5. Learning to Cope with Persisting Symptoms a. *Establishment of New Habits Affecting the Health of Mind and Body* b. *Social Support and Long COVID Knowledge Sharing*	+ +
9	** Less than 6 months (1) ** 7–12 Months (3) ** 13–18 Months (1) ** 19 + Months (8) ** Not reported (5)		1. The Landscape Behind the Long Covid Experience a. *Centrality of Physical Symptoms* b. *Navigating ‘Experts’ and the ‘True Colours’ of Personal Communities* c. *A Rollercoaster of Psychological Ambiguity* d. *Sensitising Mechanism: The Hope‑Acceptance Relationship* 2. The Everyday Experience of Participants’ Long Covid a. *Seeking Re‑Assurance and Knowledge* b. *Developing Self‑Awareness of Symptoms Through Monitoring* c. *Trial‑and‑Error of ‘Safe’ Ideas* d. *Building in Pleasure and Comfort* e. *Prioritising ‘Me’*	+ +
10	“Start of Illness”: February 2020 (2) March 2020 (1) April 2020 (1) September 2020 (1) March 2021 (3) November 2021 (1) January 2022 (1)		1. The COVID-19 Pandemic Context 2. Changed Understanding of Life 3. Lost Sense of Self 4. Understanding and the Understanding Other 5. Struggling with Acceptance 6. Sources of Meaning in Life* *Explored in various ways	+ +
11	(*) 2020 (10) (*) 2021 (6) (*) 2022 (5)		1. Loss of Physical Health 2. Loss of Mental Health 3. Loss of Social Support & Connections 4. Loss of Roles in Family 5. Loss of Self-Identity Findings additionally mapped onto Kübler-Ross’ (1969) Stages of Grief Model	–
12	Date of COVID hospitalisation (no. of participants): April 2020 (4) June 2020 (1) October 2020 (2) November 2020 (4) February 2021 (1)		1. Existential Crisis a. *Facing Psychological Threat* b. *Seeking Legitimisation* c. *Forging a Path Through Uncertainty*	+ +
13	(*) March 2020 (5) (*) April 2020 (3) (*) September 2020 (1) (*) October 2020 (1) (*) December 2020 (2)		1. Long COVID Suffering a. *Long COVID Suffering* b. *Understanding Long COVID Suffering* c. *Organisational Validation of Long COVID Suffering* d. *Societal Validation of Long COVID Suffering* 2. Identity a. *Identity Struggle* b. *Identity Work* c. *Managing Through Identity Transformation* 3. Belongingness a. *Work Group Belongingness* b. *Workplace Belongingness* c. *Employment Belongingness* d. *Long COVID Community Belongingness* e. *Long COVID Staff Support Group Belongingness*	+ +
14	“Long Covid defined as symptoms of Covid-19 lasting longer than the acute 4 week period of initial infection” (page 4)		**Overarching Theme:** Resolution of the Dichotomy **Transcending Theme:** Tension Between the Professional Expertise and Personal Experience of Long COVID Creates a Dichotomy 1. Living in Uncertainty a. *Fear of the COVID-19 Pandemic, of the Unknown Cause & of the Future* b. *Lack of Treatment Options* c. *Ambiguous Identity* 2. Why Should I Trust You if You Don’t Believe Me? a. *Broken Trust with Medical Professionals* b. *Isolation from Social Networks* c. *Broken Trust with Body* 3. Once I Know the Cause, People Will Believe Me a. *Self-Educated Through Research* b. *Medical Focus on Aetiology & the Need to Prove Illness* c. *Initial Rejection of Psychology’s Role in Long COVID*	+ +
15	Data not reported		1. Living Situation and Difficulties in Daily Activities 2. Occupational situation 3. Support from the Work Environment 4. Role Conflicts, Identity Crises, and “New Reality with Long COVID”	+ +
16	Time elapsed (in months) since acute infection: 10 (3 participants) 11 (1 participant) 12 (6 participants) 18 (5 participants) 20 (1 participant) 24 (2 participants)		1. Having an Unknown Chronic Illness a. *Reactions* b. *Denial* c. *Physical and Mental Impacts* 2. Living with Uncertainty a. *Variable/unpredictable symptoms* b. *Frustration* c. *Loss of Identity/Sense of Purpose* 3. Regaining Control a. *Advocating for Myself* b. *Mastering the symptoms/self-management* 4. Moving Forward a. *Acceptance* b. *Re-evaluation* c. *Re-defined Sense of Recovery*	+
17	Interviews took place on average 232 days (range: 96–377 days) post-COVID-19 infection		1. Realising Disruptions 2. Identity and Role Challenges 3. Reconciling Illness & Identity	+
18	(*) March 2020 (10) (*) April 2020 (3)		1. Making Sense of Symptoms 2. Feeling Let Down 3. Using Medical Knowledge and Connections 4. Wanting to Help and Wanting to Be Helped 5. Becoming a More Empathic Doctor	+/–
19	Data not reported		1. Understanding Who Helps Patients Manage Symptoms a. *Available Support Options* b. *Family Interdependence* 2. Daily Activities and the Impact on Quality of Life and Health Status a. *Changes in Functional Ability and Physical Activity* b. *Pacing as an Activity Management Strategy* c. *Post-Exertional Symptom Exacerbation* 3. Emotional Impact of Long COVID Symptoms on Personal Identity and Recovery 4. The Effect of Turbulent and Episodic Symptom Profiles on Personal Identity and Recovery	+/–
20	(*) December 2019 (1) (*) January 2020 (1) (*) March 2020 (2) (*) April 2020 (1) (*) May 2020 (1) (*) June 2020 (3) (*) October 2020 (2) (*) November 2020 (2) (*) December 2020 (1) (*) January 2021 (3) (*) April 2021 (1) (*) No data (1)		1. Crafting Normalcy 2. Affirming Identity Anchors 3. Maintaining and Using Communication Networks 4. Putting Alternative Logics to Work 5. Foregrounding Productive Action Whilst Legitimising Negative Feelings 6. Critiquing and Resisting the Status Quo	+ +
21	** 1–2 Months (15) ** 3–5 Months (29) ** 6–9 Months (26) ** 10 + Months (99)		1. Long COVID Symptoms Are Numerous and Wearing a. *Symptoms are Numerous* b. *‘Common’ Long COVID Symptoms* c. *Symptoms Vary in Presentation and Intensity* 2. The Effects of Long COVID Are Pervasive a. *Modified Sense of Self* b. *Changed Capacity to Manage Roles and Responsibilities* c. *Changed Capacity to Work* 3. Physical Activity is Difficult and, in Some Cases, Not Possible 4. Asking for Help When Few Are Listening, and Little is Working a. *Support Received* b. *Few, if Any, Treatments Work*	+ +

Alphabetic letters represent subthemes and secondary categories

### Data extraction strategy

The principal investigator (HH-M) commenced data extraction on 30th May 2025. Twenty-one individual data extraction forms (DEFs) were completed, one for each article, gathering essential data from every paper, i.e., research aims, recruitment settings, participant demographics, methodologies, findings, limitations and conclusions – for an example, see Appendix C (SM). The DEFs were subsequently reviewed by the second author (FL), ensuring a consensus was reached that sufficient detail was extracted.

### Data synthesis and presentation

Prior to commencing data synthesis, this project was registered with PROSPERO, the International Prospective Register of Systematic Reviews, under ID: CRD420251008410 (see Appendix D, SM). Research ethics declaration (Appendix E, SM) and risk assessment (Appendix F, SM) documents were completed.

To produce an overarching story across articles’ findings, synthesising varied qualitative methodologies, frames of reference, focuses and reporting styles, a Narrative Synthesis (NS) [55]. An iterative process of reading and rereading texts, exploring the use of phraseology, diction, and meaning, enabled textual descriptions of every article to be established. Next, a preliminary synthesis of findings was outlined by generating rudimentary themes and subthemes. Each article’s original themes were documented, tabulated and ‘vote counted’, i.e., frequency counting to identify descriptive patterns of a data set by similarly grouped themes/findings. The primary researcher and first author’s (HH-M) initial themes and subthemes were then ‘vote counted’, to better understand unfolding relationships across the data and refine final themes. Lastly, examining the methodological quality of included papers facilitated understanding of the robustness and trustworthiness of the synthesis. This is determined by evaluating the quantity of data, the overall quality of individual articles, a between-article analysis of the relationship across papers (assessing the strength of evidence upholding the synthesis’ conclusions), and the extent of information available to judge included articles against the inclusion criteria.

### Primary researcher’s position

This NS was conducted by the principal investigator and first author (HH-M) – a White British female from a northern city in England, and a clinical psychologist providing psychological support to LC patients through a publicly-funded Post-COVID Assessment Service. Whilst HH-M possesses professional knowledge relating to the subject matter, this author does not have lived experience of LC.

Attention was paid to preconceived ideas and potential biases, to mitigate their impact upon understandings drawn, throughout the review process. This was achieved through regular supervision, and reflective journalling, to heighten awareness of these issues and assist a continued cycle of reflexivity. HH-M remained cognisant of her ethnic and cultural background as potential influences upon interpretation and sense making. Related considerations included preconceptions and assumptions about responses to LC on a societal, political, and individual level. The context of LC care within the National Health Service (NHS), and across different healthcare systems, also required contemplation when reviewing an international body of literature.

These measures aimed to reduce researcher bias. However, the first author’s position is that personal experiences and belief systems cannot be wholly contained or removed from data synthesis and analysis procedures, a sentiment shared by many non-positivist researchers [56].

## Results

### Study quality assessment

The search strategy yielded 1,455 papers. After screening, 21 articles met eligibility for synthesis. Heterogeneity was evident in topic focus across the data set (see Appendix G, SM): three studies specifically aimed to explore self-identity in the context of LC [17, 42, 43]. All studies were qualitative, suited to their respective aims, and relatively homogeneous in methodological approach – variations of Thematic Analysis were most observed (*n* = 15). Other methodologies included Interpretative Phenomenological Analysis (*n* = 3), Content Analysis (*n* = 1), Constructivist Grounded Theory (*n* = 1), and Narrative Analysis (*n*= 1). Two studies adopted a focus group format over one-to-one interviews [31, 34]. Articles were published between 2021–2025 and originated primarily from the UK. The United States of America (USA), Germany and the Netherlands comprised the remaining countries of origin. Two UK studies recruited participants living elsewhere – Humphreys et al [41] included one participant residing in the US, similarly, Collier and Garip [28] described one participant living in Poland. Wurz et al [30] recruited participants internationally and cited several countries of residence: no data (6 participants), the UK (*n* = 67), Canada (*n* = 63), the US (*n* = 26), ‘other Europe’ (*n* = 6), and Brazil (*n* = 1).

Collectively, across studies, 613 participants were recruited. Of them, 465 identified as female, 134 identified as male, 6 identified as non-binary, one participant preferred not to answer, and one paper lacked gender descriptors for 7 participants [7]. All but one study [41] predominantly recruited females. Included papers’ sample sizes varied, ranging from 9–169 participants, with a mean of 29.19, and a mode of 18. Table 3, below, offers in-depth descriptors of all articles.

### Methodological quality

Fulfilment of the quality appraisal process evidenced good methodological quality overall. The highest quality rating (+ +) was attributed to 13 papers (61.9%) and the mid-level rating (+) applied to 5 articles (23.8%). Two studies fell between the mid-level and low-level rating categories and were therefore assigned a combined rating (+/–). Only one study met the criteria for the lowest rating (–) (see Table 3). Lower quality ratings tended to reflect combined limitations, e.g., unclear descriptions of LC, time since symptom development, patient and public involvement and engagement (PPIE), ethical considerations, researcher positionality, and minimal justification for sampling strategies or methodological approach. The discussion below details further strengths and critiques.

#### Ethics approval

Most papers explicitly stated that ethical approval was obtained. However, two articles omitted discussion regarding ethical approval [17, 54]. Deliberation of ethics is fundamental to research [57]. Thus, inadequate transparency obstructs understanding of how ethical considerations and principles were approached and upheld.

#### PPIE involvement

Many funders and ethics review committees expect embedded PPIE within research; PPIE is a recognised essential pillar of good research practice, promoting collaboration and driving relevant outputs [58, 59]. However, ten studies in the data set did not evidence PPIE or provide justification for its absence. PPIE or lived experience advisory groups reviewed data interpretations in three studies, and in a fourth study where the group additionally reviewed participant-facing documents and advised on recruitment. Three studies refined themes/categories through member checking/participant feedback, and two separate studies included patient co-authors. Thomas et al [29] shared findings with two ‘patient representatives’, and Taylor et al [36] liaised with post-COVID support group members during study planning. See Appendix H (SM) for further information.

#### Researchers’ positionality & reflexivity

Researcher reflexivity, and recognition of positionality, represent hallmarks of good practice in qualitative research [60]. Researchers carry their own personal and professional histories, identities, biases, assumptions, values, and beliefs – each capable of shaping interactions with, and understandings of, research [61]. Appreciation of a researcher’s approach to identifying and responding to this knowledge provides valuable insight [56]. Despite this consensus, insufficient exploration of researchers’ relationship to participants and targeted topics existed within the data set. Examples include isolated discussion of professional background or authors’ research experience, absent reporting of researchers’ background and positionality, and exploration of epistemological or ontological viewpoints alone (detailed overview in Appendix I, SM). Instances of vaguely referencing a commitment to reflexivity, without elaboration of examples, were also noted – Braun and Clarke [62] urge researchers to ‘go beyond’ surface-level statements.

Comparatively, Kennelly et al [34] offered a researcher team positionality statement including each author’s educational/professional background, gender, ethnicity and LC personal experience (or lack of). Petker and Ogden [53] mirrored this approach, additionally describing measures taken to minimise potential biases. Through supplementary material, Wurz et al [30] matched this information. However, philosophical underpinnings were solely discussed in text.

#### Interview durations

Mixed interview durations, not applicable for two studies [29, 30], were described. Two articles omitted interview-duration definitions [28, 43]. The remaining 17 papers reported a range of timings: interviews lasted between 17 and 210 min, with an average minimum duration of 33 min and an average maximum duration of 69.3 min. In shorter interviews, e.g., 17 min, breadth and depth of data exploration may have been negatively impacted due to constrained timings – without justification. Only two studies’ rationales for interview length/structure related to LC symptoms. Humphreys et al [41] limited interviews to 45 min to reduce cognitive burden for participants. Similarly, Hunt et al [42] interviewed across multiple sessions, if requested by participants, to minimise fatigue.

#### Illness descriptors

Heterogeneity was evident in descriptors of illness onset and LC status. Most studies detailed one of the following: the month and year of initial COVID infection, time spent living with LC, time in months since acute infection, dates of COVID hospitalisation, or a general illness-duration description. However, three studies did not report any such data [29, 32, 37]. Thompson et al [54] provided descriptions for all but one participant, Leggat et al [23] excluded descriptions for 5 participants, and Kennelly et al [34] omitted this data for 6 participants. One study merely described a broadly accepted definition of LC [53], without commenting on individual illness durations. Consequently, for some studies, it was challenging to appreciate stages of adjustment to LC and the extent of LC lived experience – aspects capable of influencing psychological perceptions of illness and ‘the self’ [63].

Most pertinently, six papers outlined descriptors of LC duration (see Table 3). Albeit some descriptions lacked precise clarity, and samples demonstrated wide-ranging divergence. Moreover, LC status (self-identified versus diagnosed) varied between samples, e.g., Twelve studies’ criteria included self-identified LC, six studies’ samples encompassed diagnosed and self-identified LC, and three papers lacked transparency regarding LC status.

#### Selection & sampling-strategy limitations

Lieberworth and Niemeijer [21], alone, offered sufficient theoretical justification for both participant selection and sampling strategy. Eight articles explicitly justified sampling strategies (see Appendix J – SM), and another provided a rationale for their participant-eligibility criteria [32]. Remaining papers lacked a stated argument for either. To some degree, each study demonstrated sampling bias. Commonly encountered issues included absent justification for specific age-related criteria (e.g., > 18 years), potential exclusion of those experiencing digital poverty, low technological literacy or illiteracy based on interview format and methods of disseminating recruitment materials, limited demographic diversity (age, ethnicity, gender), small sample sizes (although apt for some methodologies), and exclusion of non-English speakers. Additionally, two papers excluded those hospitalised for COVID-19 without explanation (see Appendix J, SM).

#### Strengths & quality summary

Notwithstanding these limitations, all but one article [43] clearly referenced research aims and selected fitting methodological approaches. Relevant literature and theoretical backgrounds were discussed, without fail. Each recruitment strategy was coherent. Means of data collection and analysis were typically rigorous and satisfactorily outlined. However, systematic record keeping was consistently unclear due to little related disclosure. Internal coherence was invariably observed, with largely convincing findings. Notably, certain studies provided limited depth and detail of participant accounts for distinct themes [29, 37, 43] or overall [20].

### Qualitative synthesis of findings

The following section comprises the NS of twenty-one primary articles. Emergent themes and subthemes, in Table 4, represent participants’ views of self-identity as shaped or altered by LC. Partial overlap in themes occurred. However, individual quotes were categorised with concepts most closely aligned to them. Mirroring original articles’ reporting, ellipses within quotations mostly represent short pauses in speech. On occasion, they represent removed text for succinctness.

**Table 4:** Major themes and subthemes

Main Themes	Subthemes	Papers Comprising Themes/Subthemes
1. Grief and Loss of the Familiar Self		7,17,20,23,33,34,35,37,41,45,46,54
2. Reflections of Self, Mirrored by Others		20,46
3. Threats to Identity	*a) Role-Related Identity Threat*	7,17,23,34,36,40,47,56
	*b) Losing Cherished Activities & Self-Narratives*	17,23,32,34,37,55
	*c) Disrupted Temporal Cohesion of Self*	20,21,34,37,47,56
6. Repairing Fractured Identity	*a) Grappling with Acceptance*	17,34,35,38,39,40,41,46,55
	*b) Identifying with Illness & Vulnerability*	17,20,34,37,41,47,54,56
	*c) Redefining & Reconstructing Identity*	17,23,37,40,41,47

#### Grief and loss of the familiar self

This theme held chief salience, reflected across twelve studies (see Table 4). For many, experiencing LC evoked a distinct absence of familiarity with the self: “I think… it’s stripped me of me” [7]. A fundamental loss of self-identity was frequently described to engender a profound sense of grief: “there's a bit of a grieving process going on for our old lives” [37]. Instances of mourning were conveyed as participants described an almost irrevocable loss of their partial or whole self-concept, “my whole image of myself is basically destroyed”; “a part of you dies a bit” [42]:

“It feels like I am mourning the person I used to be. It feels like, when I had COVID, even though I did not die, a part of me did. It’s taken a part of my life with it… It’s almost like you are in a prison in your mind. Like you are there but just can’t get out. I feel like it all the time because I want to do things…” [33].

The latter quotation demonstrates psychological distress, feeling trapped, arising from acknowledging and experiencing an inability to connect with a long-held self-identity.

Others experienced discomfort, and hinted towards depression, related to unfamiliarity with a changed self-concept, “I’d lost all sense of surety of myself… that’s not a very comfortable feeling still. I do struggle with that… I do wonder, will I come back again?” [52], “I feel like a mere shadow of the person I was before COVID-19 and that is very saddening” [30], “it’s just been very depressing, feeling like you’re never going to get better or be back to yourself” [20].

Reduced emotional sensitivity towards the suffering of others, following LC, also represented a departure from the known self, “I was made aware that I now have very little compassion or empathy – I’m not the same person. I just know I’m not me anymore” [29].

Finally, cognitive changes associated with LC diminished familiarity with a recognisable sense of self, “it’s made me a different person… my brain works differently” [52].

#### Reflections of self, mirrored by others

Across two papers, the struggle of individuals with LC to be recognised as ‘the same person’, by others, is highlighted. Difficulty was described in witnessing and adjusting to others’ reactions, with potential implications for personal understandings of self-identity. Perhaps retention of a continued sense of self is weakened when others signal that identity losses have occurred:

“I was very much the instigator and the organiser of my friendship group and now I’m not because I just don’t have the capacity. So, it’s really hard because our lives are so different now… the gap is just so huge… They’ve tried very hard and been very supportive. But I’ve also had them get very upset when they see me and say, ‘I miss my friend’. And that’s hard because I’m here, I’m still here. ‘You are talking to me, but you’re telling me you miss me’. So, they’ve had to get their head around it and kind of change the things that we can do… it’s just a lot of learning and adjusting for everybody” [42].

(My mother was) “grieving for me even though I’m still here, because it’s the loss of the person that I was. She didn’t expect to see a 30‐year‐old coming home and caring for her and seeing me in a wheelchair for the first time. It’s all been a lot to process” [42].

Here, loss of an ‘independent-adult identity’ is described and replaced with retrogression – disruption to the typical stages of life transitions, with a young adult reflecting that through their mother’s eyes they are returning to a state of dependency on parental care.

Specific elements of identity were also directly influenced by external perceptions of self. This manifested as a perceived loss of certain attributes, “I sleep a lot, or I’m in bed, or not as much fun. And not as active… so that’s hard on them because I really was like, I was like the really cool grandma” [20]. A broader sense of mirrored identity loss was detailed, necessitating acceptance of the ‘current self’, “they kind of lost the mom that they were used to. A mom that was active and was always involved and doing things. And we’re all learning to try to accept where I’m at” [20].

#### Threats to identity

Recurring narrations of threatened identity unfolded across three predominant subthemes. Collectively, these subthemes emphasise the importance of how individuals locate themselves within, and identify with, specific roles, meaningful activities, and a perceived continuity of self across time – key facets of identity, markedly disrupted by LC. In the absence of these identity anchors, participants described diminished confidence, self-worth, and hope. For some, existential angst and cognitive dissonance followed. As established self-image, self-esteem, and self-knowledge were increasingly called into question, adverse shifts in broader self-concept occurred.

#### Role-related identity threat

Termination and alteration of roles were experienced as identity threats, detailed across eight articles. Participants described self-identity drifting when confronted with reversed roles, e.g., from ‘caring’ to ‘cared for’, necessitating psychological adjustment, “I do feel a little bit lost because I just don’t know. I was a carer and now I’m being cared for. I’ve had to change, and I’ve struggled coming to terms with it a little bit” [7], “…there’s been a big change of identity. I’m a recovering patient from COVID rather than a meritorious consultant” [7].

Examples of existential angst, prompting questions concerning life’s meaning and purpose, were observed when professional and social roles were unfulfilled:

“Before I had it [LC], I was working as a social worker, two young children, physically active, I was involved with various groups and, and suddenly I could do nothing.

None of that. So, everything, everything by which I defined myself was taken away… I was left with who am I? What is the meaning of life? You know those big questions…” [43].

Diminished confidence often accompanied loss of a professional role, which fuelled the urge to withdraw from others. Consequently, the ‘current self’ was described to pale in comparison with the ‘old self’:

“I always was a fairly confident person, always had big responsible jobs… I sort of thrived on stress and all the rest of it. Now, I just feel like I’m a little old lady. I don’t have any confidence at all. I would much prefer not to go out the house at all, I’ll stay at home and cuddle up and when I’m tired go to sleep… I’m just a shadow of who I used to be, I’ve gone very withdrawn” [43].

Frequent discussion of relational roles emerged. Descriptions alluded to fixed beliefs about the duties and expectations assigned to relational roles. As LC interrupted or reconfigured role-specific norms within family units, cognitive dissonance, self-criticism, and lowered self-worth were encountered:

“I no longer feel like a main pillar in the family. I am the father, biologically in any case, but at the moment I feel pretty worthless… of course, that’s not true, I know that, but you just don’t feel like a good partner and father anymore if you can’t be the way you want to be and would like to do that with your family. You’re just not the same anymore” [32].

#### Losing cherished activities & self-narratives

Six articles illustrated the significance placed on cherished activities in the narrative construction of identity. Due to individuals with LC forgoing AoDL and hobbies closely interwoven with sense of self, deep sadness and unfavourable self-comparison (‘former self’ versus ‘current self’) occurred.

For example, “I miss [breaks down crying] I’ve missed the old me, sometimes. I miss. I miss going to the gym, going swimming. I miss running. Sorry [crying], I don’t normally get upset… I miss the old me” [32], “there was a loss of anything I might say about myself to someone—“I’m someone who likes walking, who likes music”—all those things I said when I described myself. A complete loss of everything that made me who I am…” [23]. Similarly, another participant alluded to a threatened self-identity through losing the ability to maintain an active lifestyle, “Long Covid has totally consumed my life. I was once an athlete… and lived a full, healthy, active life, now I am out of breath during a fast-paced conversation at times” [30].

Normality encountered through engagement with AoDL and daily routines was missed and contradicted understandings of selfhood, “mentally [I’m] very down. Sometimes very, very weepy, very weak just because it’s not me. I know it’s not me... You know where I used to make lunch and supper, I can just make supper now” [53].

When participation in AoDL remained possible, marked adjustments were necessitated. These revisions threatened sense of self, accentuating the lack of ‘sameness’ with former understandings of self-identity, “I’m not the same person I was… last year. I might not be able to celebrate [at my friend’s wedding] in a way I would normally be able to because of my energy levels” [53].

#### Disrupted temporal cohesion of self

Six papers illustrated the problematic nature of LC causing disrupted temporality in relation to self-identity. LC ruptured continuity of participants’ narrative thread linking sense of self across the past and present. Similarly, projections of the ‘future self’ lacked clarity and became poorly defined. Sadness, frustration, and hopelessness were consequently elicited.

Here, diminished relatability with past understandings of the self is clear, “I feel like a mere shadow of the person I was before COVID-19 and that is very saddening” [30]. Ambiguity surrounding present-day interpretations of personal identity then arises, “I don’t know this person I have become since COVID-19” [30]. Finally, difficulty envisaging reconnection with a familiar self in the future occurs:

“…I do often wonder if I will ever get back to what I used to be. It is taking such a long time, and it does frustrate me sometimes. I have not come to terms with the long COVID yet. I would just like to get back to some of what I was like before” [33].

The latter quote suggests that time itself is experienced as protracted through a longing for reconnection with continuity of self, resulting in frustration. This experience is reinforced by the absence of a defined end to this state of suspension, in turn decreasing hope, “I don’t see an end in sight. … I’m kind of out of hope for that, and I’m looking around with the way that I live now, which is, I’m a shell of the person that I used to be” [20], “because you see no end to it! … like all illness there comes a point when you can’t remember what it was like to be well, you just can’t…” [43].

The dynamic nature of identity reappraisal, necessitated by LC, may further displace present and future-projected understandings of self. One participant reflected on identifying as a ‘COVID long hauler’. However, over time, they appeared to question or mistrust this identity as alternative illness identities were considered, and understandings of LC continually evolve. The ‘COVID long hauler’ identity therefore lacked permanence, further compounding the threat of disrupted temporal self-identity:

“I think I definitely identify as a COVID long hauler. So, I have been kind of starting to… ask myself and kind of ask others around me, ‘At what point – basically at what point am I not a COVID long hauler, but instead I’m someone who has dysautonomia and POTS?” [54].

#### Repairing fractured identity

Reports of LC triggering various identity fractures, requiring repair, were prevalent. This theme tackles issues of accepting a revised sense of self, updating beliefs about the self, identifying with illness, and tackling identity reconstruction.

#### Grappling with acceptance

Nine papers recounted stories of grappling with acceptance of an altered identity. Acceptance of identity changes were viewed as a major psychological feat: “It’s also been a change for me to recognise that I’m never, might never, be the same as I were before. …That’s a really big psychological thing to get over” [35]. Hence, narrations of oscillation between acceptance and struggle materialised, “it’s a struggle… of just knowing… your old capacity and not just being able to fulfil that, and it’s still hard… I mean, obviously now, I’m accepting my new self-more, but it’s still hard sometimes…” [42]. However, acceptance was too elusive for some, “I just could not get over the fact I wasn’t getting better, I could just see myself as an ill person. I just could not accept it…” [43].

Others were continuing to strive towards acceptance, “if this is my new me, I’m learning to live with it… I can’t do much physically and I can’t do much cognitively… coming to understand that right now… this is my normal” [31]:

“I think the hardest thing for me, and for others that I've spoken to, is the acceptance that something is potentially changed as ongoing and that we need to find a new version of ourselves…we're giving up our old selves and having to become somebody completely new” [37].

Contrarily, narrations demonstrating acceptance of current self and LC-related limitations transpired, “I’ve come back quite openly saying, I’m not the person that went off. I’m not capable of everything I did before… But there is lots I can do. And I’m here” [35].

#### Identifying with illness & vulnerability

Between eight studies, reformulating selfhood to encompass illness identity was described. However, occurrences of rejecting identification with illness were also documented. Dichotomies of healthy versus ill, indestructible versus vulnerable, were addressed within this subtheme.

Incorporating an illness-identity label, e.g., COVID ‘long hauler’ – someone living with LC, into a broader self-identity could legitimise and validate experiences of LC, “(I) embraced being disabled as a part of my

 identity” [54]:

“It’s so funny because I remember telling people I still had symptoms. And, a girlfriend of mine was like, “Oh, you’re a long hauler.” And, I’d never heard of that… then I went home and did research. Yep, that’s definitely what I am” [54].

However, one participant felt unable to reconcile emotional and logical components of reasoning for identification with ‘long-hauler’ status. For example, despite ‘qualifying’ for recognition of this illness identity, one participant described reluctance to proudly own such a label due to it encompassing a wide spectrum of illness-severity experiences. The avoidance of shame is alluded to, by exercising caution regarding ‘long-hauler’ labelling:

“I technically fit the description because I am 13 months out and I still have symptoms of COVID. But at the same time, I am hesitant to just label myself that and proudly wear that label because there are people who have been in the ICU for months, and we’re not the same... they have experienced something so much more severe than I have” [54].

Others placed significance on avoiding internal or external recognition of illness identity, “I feel like a different person. But on the outside, I try to be the same person. I tend not to explain to so many people, because I do not want to classify myself having a chronic disease” [17]. Evidence conflicting illness-rejection narratives were therefore confronting and difficult to concede:

“I feel I struggle with not being well, and I don’t want to be a burden. I want my old life back. …It was so hard for me to ask for that (accessible parking placard) because part of that was admitting that I was sick enough that I needed it” [20].

Differentiating, and distancing, the self from LC-illness identity may occur due to diverse LC symptomology, i.e., when one cannot relate to predominant LC narratives, or perhaps as a defence to deny acknowledgement of loss, grief, and identity alteration, “I haven’t found it particularly useful to be in groups with other long COVID people. I’m on that Facebook group but I don’t identify with that group, I suppose” [52].

Yet even identification with an illness-identity provoked further modifications of personal identity. For example, letting go of self-narratives of invincibility whilst acknowledging one’s fragility. This realisation generated a renewed gratitude for health:

“From… being young, I thought I was like untouchable, and I would never get ill, so it kind of taught me that we are all vulnerable in a way or health is valuable and we shouldn’t take it for granted” [33].

#### Redefining & reconstructing identity

Throughout six articles, the need for, and experience of, redefining or reconstructing self-identity was explored.

Across multiple accounts, LC experience catalysed a process of refining elements of self. The example below details learning taken from the participant’s dual position as a clinician and LC patient, characterising the future redefined self as more empathetic and aware of personally-held prejudgements:

“It wasn’t an active prejudice, but in the back of my mind I hadn’t thought about it… a number of us on the group have said how ashamed we are of some of the attitudes we’ve had towards people, and lack of empathy… this concept of being irritated by patients when they’re not really pleased when something comes back normal… hopefully it will make me a better and more empathetic doctor at the end” [36].

Evidence of PTG manifested further in some participants’ reflections. Unsustainable means of living were recalled, alongside reports of recalibrated life priorities, which fostered balanced living and enhanced understanding of others and the self:

“If I hadn’t had this time of reflection, I just would have carried on, we all would have just carried on working… and I think I’m probably in a better place because I haven’t worked. I’ve had time to share with my family, cos they’ve all been here. I think I’ve got a greater understanding of myself and of everybody” [43].

“I’ve changed a lot as a person. So earlier, it was go up the career ladder, follow this thing, be on all the time, deliver, get that next promotion. And that was my life, it has always been that way. And this [Long COVID] has made me think what is more important in my life, and how not to take health for granted” [17].

A number of participants recounted shifts in life priorities which strengthened appreciation for seeking greater acceptance of current identity:

“What I have learnt looking back now, before COVID, I thought I should never take a break from work or my routine. I almost thought that If I stopped then the world would stop. I now realise that the world does not stop if I stop, and that I need to take priorities and manage my time differently. I also need to accept myself as I am, and I am working on it” [33].

Uncertainty is fuelled by numerous aspects of the LC experience, subsequently provoking the difficult task of tolerating it. Yet, for some, uncertainty in relation to self-identity became a known and embraced position. In fact, the notion of an ‘unwritten’ self-identity was potentially exciting, an “adventure”, signalling appreciation of a transforming life and self:

“Just write ‘I am’ and leave a gap, and just let that gap be. Let that uncertainty be. I don’t know who I am exactly now, but ‘I am’ is enough. I have that written on my fridge. The end of that sentence is an adventure and doesn’t have to be what it was before. It’s probably not going to be everything it was before because I will make some changes even if I get 100%. After that, I feel more acceptance that life has changed” [23].

## Conclusions

This systematic literature review provides the first overarching narrative of findings detailing identity alteration through LC lived experience. Results highlight the multifaceted aspects of self-identity, many of which are evidently challenged and revised through experiencing LC. Stories of retrogression, stagnation, and growth transpired, as themes of loss, grief, threatened self-concept, and identity redefinition were explored.

Most notable within this NS was common experiences of discomfort, sadness, and painful grief accompanying loss of familiarity with sense of self – provoked by LC. When known self-identity was perceived to be ‘destroyed’, described most strikingly as ‘dead’, mourning occurred. When connection was partly maintained with familiar aspects of self, internally, but this did not correspond with an ability to enact routine behaviours, externally, participants described a trapped sense of self. This subsequently reinforced loss and distance from the known self, stressing the important role of AoDL and hobbies in upholding and reinforcing favoured self-narratives. Moreover, participants attested to loss of ‘sameness’ of self through the direct experience of LC symptoms, e.g., neurocognitive changes and functional losses. Feasibly, the ongoing embodied experience of LC may constitute a continuous reminder of identity deviations – a notion documented in apposite research [18, 30].

Closely following the dominant narrative of identity grief were stories of grappling with acceptance of an altering self-identity. Findings revealed that individuals living with LC often oscillate between struggling against and embracing a ‘new’ self, described to be psychologically taxing. Participants discussed ‘learning to live with’, and accept, altered cognitive capacity, new limits of functional ability, and uncertainty concerning reacquaintance with the familiar self. In the context of navigating grief, applicable to LC-related identity loss, the occurrence of such oscillation is well established. Within grief literature, this is referred to as the ‘Dual Process Model of Coping’ [64] whereby individuals switch between loss-orientated and restoration-orientated focus. Whilst this model primarily relates to bereavement following a person’s death, the underlying premise arguably holds utility for understanding grief-loss narratives more broadly – supported by this NS.

Moreover, the NS identified recurrent challenges in assuming or rejecting illness identity. Among participant accounts, a struggle to identify with illness and actively engage in identity reconstruction was recognised. Personal meanings participants attached to LC-illness identities, e.g., COVID ‘long hauler’, were influential in assisting or preventing assimilation of them into sense of self. For example, several participants felt recognised and validated by adopting a label representative of their experience, others believed that their illness severity did not ‘qualify’ for long-hauler status, or the description simply did not resonate. Labels attached to chronic illness hold strong connotations that some wished to remain distant from. Additionally, identification with illness was unsettling; it frequently meant dropping former understandings of self, e.g., self as invincible. It has been argued that identification with chronic illness demands a reflexive renegotiation of self-concept, evolving over time [65, 66]. However, the novelty of LC illness and unpredictability of fluctuating symptoms, combined with an unknown illness-trajectory, may further obfuscate this process – exemplified in one participant’s description of LC-illness identity seeming dynamic and impermanent. Self-identity can clearly shift in the context of adjustment-illness narratives, initiating a highly complex and uniquely personal experience.

Role-related identity crises surfaced as an equally common narrative thread. Role reversals, e.g., ‘caring’ to ‘cared for’, required substantial psychological adjustment and were closely linked with self-identity alteration. Participants discussed questioning their entire identity in circumstances where they ceased to fulfil roles holding significant personal value. Similarly, the need for adjusted performance within relational and professional roles was evident but frequently difficult to action. As illustrated in the broader context of chronic-illness literature [67, 68], this posed a significant threat to selfhood. Resultantly, participants spoke of reduced self-worth and greater self-criticism. Additionally, accounts hinted to experiences of cognitive dissonance, e.g., discomfort borne of a misalignment between behaviour and personal values or beliefs about what is required within specific relational roles.

PTG was present in certain participants’ narratives of identity redefinition. However, not all participants were engaged in a process of active identity reconstruction prior to interview. Similar themes of PTG exist within research exploring ME/CFS – a condition with many parallels to LC [69]. In the current review, participants referenced an improved outlook on life and discussed reimagining their self-concept and identity through multifarious means: recognition of unbalanced ways of living, gaining depth in understanding of themselves and others, enhanced appreciation of previously taken-for-granted experiences, reprioritising life goals/values, and embracing acceptance of the unknown. Instances of envisaging the future, with opportunities for reconceptualising sense of self, were occasionally framed as an exciting prospect offering adventure. Time spent living with LC may significantly determine the nature of such descriptions. However, those provided here are representative of varying illness durations across a wide spectrum of experience.

All but one theme mapped closely with original articles’ themes/subthemes, indicating the strength of common narratives across germane literature published to date. “Reflections of Self, Mirrored by Others” emerged as a novel theme. However, this focus represents a niche aspect of experiences altering self-identity – one distant from broader conceptual focuses spanning the original data set. Through this theme, it can be readily appreciated that personal experiences of LC, and identity construction, do not exist in a vacuum. Both are subject to the influence of external factors. Hence, familial and societal responses towards an individual’s experience of LC were indicated to shape understandings of self-identity. Existing research reflects the notion that self-concept is, in part, socially constructed [66]. In a highly stigmatised condition, some individuals with LC may consequently engage in impression management by hiding the impact of their condition to protect self-identity [9].

### Limitations

Qualitatively-orientated LC research remains in its infancy and continues to evolve. From this perspective, the current review is characterised as an introductory exploration of literature detailing LC’s influence upon self-identity; particular topics were not addressed in the entirety of depth warranted, due to limitations on data availability in the primary research. Although the data set contained themes strongly aligned with this review’s aims/scope, many extraneous themes and subthemes were also present, (see Appendix G, SM). Particular articles’ participant quotes were therefore referenced more frequently than others. Additionally, the second main theme, “Reflections of Self, Mirrored by Others”, was derived from only two papers (see Table 4). However, it was deemed to carry sufficient weight over several distinct accounts which justified inclusion.

Inadvertent, and in some instances deliberate, exclusion of certain participants’ experiences occurred through studies’ vague or unjustified inclusion and exclusion rationales. However, the first author acknowledges an extension of this issue in the current review through limitations placed on non-English language papers. Inevitably, the findings described are limited to the context of Western culture; therefore, generalisability cannot be assumed. Moreover, participant samples lacked diversity and were predominantly comprised of white females. Heterogeneity of studies’ participant inclusion criteria (e.g., self-diagnosed vs. formally diagnosed LC) may also have captured broader non-LC experiences through errors in self-diagnosis, impacting the reliability of the synthesised findings. However, errors in formal diagnosis, reflecting limited definitive diagnostic tests, are equally possible and hold similar implications for data reliability.

Whilst most articles (13) originated solely or partly from the UK, varied geographical origins were observed; context must be considered when reflecting on findings. For example, within the UK’s primary healthcare system, the NHS, disparity exists in access to services with some regions lacking LC-specialist clinics [70]. Many more established LC clinics are no longer funded and operational [71]. Similar difficulties are mirrored in other countries’ healthcare systems [72]. Participants’ perceptions of LC upon self-identity may therefore have been shaped by disparate access to tailored support, aiding management and understanding of the condition – especially psychological therapies. Yet, reporting across the data set did not enable an evaluation of correlation between PTG (for example) and access to service-led support.

All findings of this study are secondary interpretations of primary data, already analysed through the lens of each article’s respective researchers. Participants’ accounts are themselves subjective interpretations of experience. Equally, themes and subthemes generated within this review reflect one author’s understanding (HH-M). The possibility that the results may have differed through a merging of perspectives (by multiple researchers bringing unique viewpoints and frames of reference to data interpretation) should be acknowledged. Although the trustworthiness of the synthesis was assessed by adhering to NS guidance [55], inclusion of one study with a sub-optimal quality rating (–) may have impacted optimal trustworthiness. The Risk of Bias Assessment Tool for Systematic Reviews (ROBIS) [73], see Appendix K (SM), further highlights potential bias in the current review. Despite the methodological-quality issues discussed, inevitably influencing the NS, the findings successfully consolidated relevant literature whilst retaining strength in dominant narratives identified.

### Implications of the NS

#### Policy & practice

Presented findings emphasise the need for psychological assessment and interventions to address identity concerns in the LC population. This should entail recognition of loss-grief narratives, acknowledging the pain of disconnection from the known self and appreciating changes in self-identity across a myriad of contexts. Exploration of this nature may be crucial prior to examining the possibility of defining and accepting illness identity; notably, fostering enhanced acceptance of an illness identity may mediate improved illness self-management [74]. In turn, this can enable a sense of mastery and control over certain aspects of illness lived experienced [75]. Psychological barriers preventing integration of LC-illness identity into broader sense of self therefore warrant consideration.

Psychological support may also encourage a re-examination of focus, meaning, and purpose in life. Establishing, and aligning with, personal values in the context of adjustment to a chronic condition might restore motivation, hope, and support positive identity transformation, particularly when paired with enhancing a fluid perspective of the conceptualised self [76, 77]. One intervention prioritising values alignment and enhancing psychological flexibility is Acceptance and Commitment Therapy (ACT), shown to yield positive outcomes in wider health-psychology contexts and recommended, for example, by NICE in the treatment of chronic pain [76, 78, 79]. More recently, ACT has been identified as an acceptable and feasible intervention for supporting the LC population [80, 81]. However, further research evaluating the effectiveness of ACT in this context is required.

In adjustment to chronic illness, excessive and negative past-to-present self-evaluations may hinder identity transformation – perpetuating a loop of self-criticism and rumination [82, 83]. Building self-compassion around LC challenges might disrupt these cycles and buffer against fusion with upward temporal comparisons of self [84]. In chronic-illness populations, a strong association is evidenced between the presence of self-compassion and lower psychological distress [85]. Self-compassion may further reduce stress, feelings of shame, and reinforce engagement with adaptive-coping strategies (including support-seeking behaviour) [86]. The potential utility of offering Compassion Focused Therapy to those with LC therefore warrants further attention and consideration [81].

A pressing need remains for additional guidance informing psychological interventions intended for the LC community. Future guidance developed by NICE and broader representative bodies, e.g., the British Psychological Society, should acknowledge the full spectrum of psychological experiences associated with LC, including loss, grief, and alteration of self-identity, with proposals for clinical practice. In rare instances of psychological care being referenced in current guidance, recommendations for clinical practice are insufficiently detailed [27]. In response, formalised requests for further iterations of relevant NICE guidance are acknowledged [87].

### Research

Greater depth of understanding should be sought to determine precisely how, and to what extent, LC patients’ self-identity alters through others’ perceptions of them post-LC development. Future research could also investigate how LC influences self-identity at specific timepoints across the illness-adjustment process. The current review was unable to determine correlations between time living with LC and the specific narratives identified. Further related research may prioritise a longitudinal approach, especially when attempting to understand experiences and facilitators of PTG – a necessary, and currently finite, area of research. The acceptability, feasibility, and effectiveness of psychological interventions encompassing identity-focused work must also be further addressed, building on the growing evidence base.

Commonalities in understandings of self-identity, shaped by LC, have been outlined. All themes touched upon the need to redefine and repair self-concept and self-identity, necessitating psychological adjustment, striving towards acceptance, and acknowledgement of loss in the process. However, disrupted temporal self-identity may be maintained through the uncertain and ill-defined trajectory of LC illness. These findings are consistent with comparable literature exploring identity shifts in those living with ME/CFS – perhaps a useful reference point for understanding the future course of identity adaptation in the LC population [67, 69, 88].

As identity reconstruction was actively embarked upon, contrasting narratives conveying a spectrum of experiences from ‘temporal stuckness’ (see Skilbeck, Spanton & Paton) [33] to PTG were illuminated. However, caution is advised when interpreting this finding; participants may fluidly shift across this spectrum, oscillating between a loss-orientated and restoration-orientated focus. Overall, this NS captures the nuance of disrupted self-identity triggered by LC.

## Supplementary Information

Supplementary Material 1

## Data Availability

Data sharing is not applicable to this article as no datasets were generated or analysed during the current study.

## Abbreviations

ACT: Acceptance and Commitment Therapy
AoDL: Activities of Daily Living
DEFs: Data Extraction Forms
LC: Long COVID
NS: Narrative Synthesis
NICE: National Institute for Health and Care Excellence
PPIE: Patient and Public Involvement and Engagement
PTG: Post-Traumatic Growth
ROBIS: Risk of Bias Assessment Tool for Systematic Reviews
SM: Supplementary Materials
UK: United Kingdom
USA: United States of America
