# Supplementary material for: Exploring the influence of long COVID upon self-identity: a systematic literature review
Source: Arch Public Health. 2026 Aug 11;84:189. doi: 10.1186/s13690-026-02017-8 (PMC13501567; doi:10.1186/s13690-026-02017-8)
Supplement: Supplementary file 1 [file 13690_2026_2017_MOESM1_ESM.docx]

**Supplementary Material**

Applied Search Terms

“Long COVID” OR “Post Covid Condition” OR “Post Covid Syndrome” OR “Post Acute Sequelae of COVID-19” OR “PASC”

AND

“Patient” OR “Survivor” OR “Individual” OR "Long COVID Patient*" OR “Expert by Experience”

AND

"Lived Experience" OR "Patient* Lived Experience" OR "Story" OR "Perception”

AND

"Self-Iden*" OR "Sense of Self" OR "Selfhood" OR "Role" OR "Identity"

| **Section and Topic** | **Item #** | **Checklist item** | **Location where item is reported** |
| --- | --- | --- | --- |
| **TITLE** | | |  |
| Title | 1 | Identify the report as a systematic review. | Included in the title |
| **ABSTRACT** | | |  |
| Abstract | 2 | See the PRISMA 2020 for Abstracts checklist. | Included within Supplementary Material |
| **INTRODUCTION** | | |  |
| Rationale | 3 | Describe the rationale for the review in the context of existing knowledge. | Included in the introduction |
| Objectives | 4 | Provide an explicit statement of the objective(s) or question(s) the review addresses. | Included in the introduction |
| **METHODS** | | |  |
| Eligibility criteria | 5 | Specify the inclusion and exclusion criteria for the review and how studies were grouped for the syntheses. | Included in the methods section |
| Information sources | 6 | Specify all databases, registers, websites, organisations, reference lists and other sources searched or consulted to identify studies. Specify the date when each source was last searched or consulted. | Included in the methods section |
| Search strategy | 7 | Present the full search strategies for all databases, registers and websites, including any filters and limits used. | Included in the methods section / Supplementary Material |
| Selection process | 8 | Specify the methods used to decide whether a study met the inclusion criteria of the review, including how many reviewers screened each record and each report retrieved, whether they worked independently, and if applicable, details of automation tools used in the process. | Included in the methods section |
| Data collection process | 9 | Specify the methods used to collect data from reports, including how many reviewers collected data from each report, whether they worked independently, any processes for obtaining or confirming data from study investigators, and if applicable, details of automation tools used in the process. | Included in the methods section |
| Data items | 10a | List and define all outcomes for which data were sought. Specify whether all results that were compatible with each outcome domain in each study were sought (e.g. for all measures, time points, analyses), and if not, the methods used to decide which results to collect. | N/A |
|  | 10b | List and define all other variables for which data were sought (e.g. participant and intervention characteristics, funding sources). Describe any assumptions made about any missing or unclear information. | Included in the methods section / Supplementary Material |
| Study risk of bias assessment | 11 | Specify the methods used to assess risk of bias in the included studies, including details of the tool(s) used, how many reviewers assessed each study and whether they worked independently, and if applicable, details of automation tools used in the process. | Broadly discussed in quality assessment - results |
| Effect measures | 12 | Specify for each outcome the effect measure(s) (e.g. risk ratio, mean difference) used in the synthesis or presentation of results. | N/A |
| Synthesis methods | 13a | Describe the processes used to decide which studies were eligible for each synthesis (e.g. tabulating the study intervention characteristics and comparing against the planned groups for each synthesis (item #5)). | Included in the methods section |
|  | 13b | Describe any methods required to prepare the data for presentation or synthesis, such as handling of missing summary statistics, or data conversions. | N/A |
|  | 13c | Describe any methods used to tabulate or visually display results of individual studies and syntheses. | Included in the methods section |
|  | 13d | Describe any methods used to synthesize results and provide a rationale for the choice(s). If meta-analysis was performed, describe the model(s), method(s) to identify the presence and extent of statistical heterogeneity, and software package(s) used. | Included in the methods section |
|  | 13e | Describe any methods used to explore possible causes of heterogeneity among study results (e.g. subgroup analysis, meta-regression). | N/A |
|  | 13f | Describe any sensitivity analyses conducted to assess robustness of the synthesized results. | N/A |
| Reporting bias assessment | 14 | Describe any methods used to assess risk of bias due to missing results in a synthesis (arising from reporting biases). | Included in Supplementary Material (ROBIS) and mentioned in the methods section. |
| Certainty assessment | 15 | Describe any methods used to assess certainty (or confidence) in the body of evidence for an outcome. | N/A |
| **RESULTS** | | |  |
| Study selection | 16a | Describe the results of the search and selection process, from the number of records identified in the search to the number of studies included in the review, ideally using a flow diagram. | Figure 1 – PRISMA diagram (results) |
|  | 16b | Cite studies that might appear to meet the inclusion criteria, but which were excluded, and explain why they were excluded. | Figure 1 – PRISMA diagram (results) |
| Study characteristics | 17 | Cite each included study and present its characteristics. | Included in Table 3 – Supplementary Material |
| Risk of bias in studies | 18 | Present assessments of risk of bias for each included study. | Discussed broadly in quality assessment  + Supplementary Materials |
| Results of individual studies | 19 | For all outcomes, present, for each study: (a) summary statistics for each group (where appropriate) and (b) an effect estimate and its precision (e.g. confidence/credible interval), ideally using structured tables or plots. | N/A |
| Results of syntheses | 20a | For each synthesis, briefly summarise the characteristics and risk of bias among contributing studies. | Discussed broadly in quality assessment  + Supplementary Materials |
|  | 20b | Present results of all statistical syntheses conducted. If meta-analysis was done, present for each the summary estimate and its precision (e.g. confidence/credible interval) and measures of statistical heterogeneity. If comparing groups, describe the direction of the effect. | N/A |
|  | 20c | Present results of all investigations of possible causes of heterogeneity among study results. | Discussed broadly in quality assessment  + Supplementary Materials |
|  | 20d | Present results of all sensitivity analyses conducted to assess the robustness of the synthesized results. | N/A |
| Reporting biases | 21 | Present assessments of risk of bias due to missing results (arising from reporting biases) for each synthesis assessed. | Discussed broadly in quality assessment  + Supplementary Materials |
| Certainty of evidence | 22 | Present assessments of certainty (or confidence) in the body of evidence for each outcome assessed. | Discussed broadly in quality assessment  + Supplementary Materials |
| **DISCUSSION** | | |  |
| Discussion | 23a | Provide a general interpretation of the results in the context of other evidence. | Included in Discussion & Implications |
|  | 23b | Discuss any limitations of the evidence included in the review. | Included in Discussion & Implications |
|  | 23c | Discuss any limitations of the review processes used. | Included in Discussion & Implications |
|  | 23d | Discuss implications of the results for practice, policy, and future research. | Included in Discussion & Implications |
| **OTHER INFORMATION** | | |  |
| Registration and protocol | 24a | Provide registration information for the review, including register name and registration number, or state that the review was not registered. | Included in the methods section |
|  | 24b | Indicate where the review protocol can be accessed, or state that a protocol was not prepared. | Included in the methods section – referenced PROSPERO ID |
|  | 24c | Describe and explain any amendments to information provided at registration or in the protocol. | N/A |
| Support | 25 | Describe sources of financial or non-financial support for the review, and the role of the funders or sponsors in the review. | Discussed (author statements) |
| Competing interests | 26 | Declare any competing interests of review authors. | Discussed (author statements) |
| Availability of data, code and other materials | 27 | Report which of the following are publicly available and where they can be found: template data collection forms; data extracted from included studies; data used for all analyses; analytic code; any other materials used in the review. | Supplementary Materials |

*From:* Page MJ, McKenzie JE, Bossuyt PM, Boutron I, Hoffmann TC, Mulrow CD, et al. The PRISMA 2020 statement: an updated guideline for reporting systematic reviews. BMJ 2021;372:n71. doi: 10.1136/bmj.n71. This work is licensed under CC BY 4.0. To view a copy of this license, visit <https://creativecommons.org/licenses/by/4.0/>

| **Section and Topic** | **Item #** | **Checklist item** | **Reported (Yes/No)** |
| --- | --- | --- | --- |
| **TITLE** | | |  |
| Title | 1 | Identify the report as a systematic review. | Yes |
| **BACKGROUND** | | |  |
| Objectives | 2 | Provide an explicit statement of the main objective(s) or question(s) the review addresses. | Yes |
| **METHODS** | | |  |
| Eligibility criteria | 3 | Specify the inclusion and exclusion criteria for the review. | No – Included in text |
| Information sources | 4 | Specify the information sources (e.g. databases, registers) used to identify studies and the date when each was last searched. | No – Included in text |
| Risk of bias | 5 | Specify the methods used to assess risk of bias in the included studies. | No – Included in text |
| Synthesis of results | 6 | Specify the methods used to present and synthesise results. | Yes |
| **RESULTS** | | |  |
| Included studies | 7 | Give the total number of included studies and participants and summarise relevant characteristics of studies. | Yes + in text |
| Synthesis of results | 8 | Present results for main outcomes, preferably indicating the number of included studies and participants for each. If meta-analysis was done, report the summary estimate and confidence/credible interval. If comparing groups, indicate the direction of the effect (i.e. which group is favoured). | Yes + in text |
| **DISCUSSION** | | |  |
| Limitations of evidence | 9 | Provide a brief summary of the limitations of the evidence included in the review (e.g. study risk of bias, inconsistency and imprecision). | No – in text. |
| Interpretation | 10 | Provide a general interpretation of the results and important implications. | Yes |
| **OTHER** | | |  |
| Funding | 11 | Specify the primary source of funding for the review. | Will be included underneath Abstract |
| Registration | 12 | Provide the register name and registration number. | Yes |

Page M J, et al. The PRISMA 2020 Statement: An Updated Guideline for Reporting Systematic Reviews. *BMJ* 2020/2021; 372: n71.

Appendix A: NICE Checklist’s Categorisation of Ratings

| Appendix B: Completed NICE Quality Checklist (Example) | | |
| --- | --- | --- |
| **Study identification:** Include author, title, reference, year of publication | **Authors:** Kalfas, M., Jolley, C., Hart, N., Rafferty, G. F., Duncan, E. L., Nicholson, T., Ashworth, M., Brewin, D., Barrett, B., Witard, O. C., Ridge, D., & Chalder, T.  **Title:** Exploring the Experiences of Living with the Post‐COVID Syndrome: A Qualitative Study  **Year:** 2024 | |
| **Guidance topic:** | Exploring the healthcare experiences and needs of people with Post-COVID Syndrome. | |
| **Checklist completed by:** | H H-M | |
| **Key research question/aim:** |  | |
| **Theoretical approach** | | |
| **1. Is a qualitative approach appropriate?**  For example:   - Does the research question seek to understand processes or structures, or illuminate subjective experiences or meanings? - Could a quantitative approach better have addressed the research question? | Appropriate  Inappropriate  Not sure  **++** | Comments:   - Does the research question seek to understand processes or structures, or illuminate subjective experiences or meanings? Yes - Could a quantitative approach better have addressed the research question? No |
| **2. Is the study clear in what it seeks to do?**  For example:   - Is the purpose of the study discussed – aims/objectives/research question/s? - Is there adequate/appropriate reference to the literature? - Are underpinning values/assumptions/theory discussed? | Clear  Unclear  Mixed  **++** | Comments:   - Is the purpose of the study discussed – aims/objectives/research question/s? Yes - Is there adequate/appropriate reference to the literature? Yes - Are underpinning values/assumptions/theory discussed? Yes – compared with similar literature in chronic pain. |
| **Study design** | | |
| **3. How defensible/rigorous is the research design/methodology?**  For example:   - Is the design appropriate to the research question? - Is a rationale given for using a qualitative approach? - Are there clear accounts of the rationale/justification for the sampling, data collection and data analysis techniques used? - Is the selection of cases/sampling strategy theoretically justified? | Defensible  Indefensible  Not sure  − | Comments:   - Is the design appropriate to the research question? Yes - Is a rationale given for using a qualitative approach? No - Are there clear accounts of the rationale/justification for the sampling, data collection and data analysis techniques used? No - Is the selection of cases/sampling strategy theoretically justified? Unclear |
| **Data collection** | | |
| **4. How well was the data collection carried out?**  For example:   - Are the data collection methods clearly described? - Were the appropriate data collected to address the research question? - Was the data collection and record keeping systematic? | Appropriately  Inappropriately  Not sure/inadequately reported  **++** | Comments:   - Are the data collection methods clearly described? Yes - Were the appropriate data collected to address the research question? Yes - Was the data collection and record keeping systematic? Data collection – yes. Unclear about record keeping. |
| **Trustworthiness** | | |
| **5. Is the role of the researcher clearly described?**  For example:   - Has the relationship between the researcher and the participants been adequately considered? - Does the paper describe how the research was explained and presented to the participants? | Clearly described  Unclear  Not described  − | Comments:   - Has the relationship between the researcher and the participants been adequately considered? No - Does the paper describe how the research was explained and presented to the participants? Not clearly |
| **6. Is the context clearly described?**  For example:   - Are the characteristics of the participants and settings clearly defined? - Were observations made in a sufficient variety of circumstances - Was context bias considered | Clear  Unclear  Not sure  **++** | Comments:   - Are the characteristics of the participants and settings clearly defined? Yes - Were observations made in a sufficient variety of circumstances Yes - Was context bias considered? Yes |
| **7. Were the methods reliable?**  For example:   - Was data collected by more than 1 method? - Is there justification for triangulation, or for not triangulating? - Do the methods investigate what they claim to? | Reliable  Unreliable  Not sure  **+** | Comments:   - Was data collected by more than 1 method? No - Is there justification for triangulation, or for not triangulating? Process described but justification not discussed. - Do the methods investigate what they claim to? Yes |
| **Analysis** | | |
| **8. Is the data analysis sufficiently rigorous?**  For example:   - Is the procedure explicit – i.e., is it clear how the data was analysed to arrive at the results? - How systematic is the analysis, is the procedure reliable/dependable? - Is it clear how the themes and concepts were derived from the data? | Rigorous  Not rigorous  Not sure/not reported  **++** | Comments:   - Is the procedure explicit – i.e., is it clear how the data was analysed to arrive at the results? Yes - How systematic is the analysis, is the procedure reliable/dependable? Yes, systematically described - Is it clear how the themes and concepts were derived from the data? Yes |
| **9. Is the data 'rich'?**  For example:   - How well are the contexts of the data described? - Has the diversity of perspective and content been explored? - How well has the detail and depth been demonstrated? - Are responses compared and contrasted across groups/sites? | Rich  Poor  Not sure/not reported  − | Comments:   - How well are the contexts of the data described? Adequately - Has the diversity of perspective and content been explored? Unclear - How well has the detail and depth been demonstrated? Not hugely detailed – lacks depth - Are responses compared and contrasted across groups/sites? No, mostly grouped together |
| **10. Is the analysis reliable?**  For example:   - Did more than 1 researcher theme and code transcripts/data? - If so, how were differences resolved? - Did participants feedback on the transcripts/data if possible and relevant? - Were negative/discrepant results addressed or ignored? | Reliable  Unreliable  Not sure/not reported  **++** | Comments:   - Did more than 1 researcher theme and code transcripts/data? Yes - If so, how were differences resolved? Implicitly suggested through multiple authors checking the codes + feedback from a PPI group (members involved in the study) - Did participants feedback on the transcripts/data if possible and relevant? Yes - Were negative/discrepant results addressed or ignored? Not explicitly stated, but somewhat described through discussion with PPI group |
| **11. Are the findings convincing?**  For example:   - Are the findings clearly presented? - Are the findings internally coherent? - Are extracts from the original data included? - Are the data appropriately referenced? - Is the reporting clear and coherent? | Convincing  Not convincing  Not sure  **++** | Comments:   - Are the findings clearly presented? Yes - Are the findings internally coherent? Yes - Are extracts from the original data included? Yes - Are the data appropriately referenced? Yes - Is the reporting clear and coherent? Yes |
| **12. Are the findings relevant to the aims of the study?** | Relevant  Irrelevant  Partially relevant | Comments: Yes, the findings are relevant to the aims. |
| **13. Conclusions**  For example:   - How clear are the links between data, interpretation and conclusions? - Are the conclusions plausible and coherent? - Have alternative explanations been explored and discounted? - Does this enhance understanding of the research topic? - Are the implications of the research clearly defined?   **Is there adequate discussion of any limitations encountered?** | Adequate  Inadequate  Not sure  ++ | Comments:   - How clear are the links between data, interpretation and conclusions? Clear - Are the conclusions plausible and coherent? Yes - Have alternative explanations been explored and discounted? Somewhat through PPI consultation - Does this enhance understanding of the research topic? Yes - Are the implications of the research clearly defined? Yes - **Is there adequate discussion of any limitations encountered?** **Yes** |
| **Ethics** | | |
| **14. How clear and coherent is the reporting of ethics?**  For example:   - Have ethical issues been taken into consideration? - Are they adequately discussed e.g., do they address consent and anonymity? - Have the consequences of the research been considered i.e., raising expectations, changing behaviour? - Was the study approved by an ethics committee? | Appropriate  Inappropriate  Not sure/not reported  ++ | Comments:   - Have ethical issues been taken into consideration? Yes - Are they adequately discussed e.g., do they address consent and anonymity? Yes - Have the consequences of the research been considered i.e., raising expectations, changing behaviour? Yes - Was the study approved by an ethics committee? Yes |
| **Overall assessment** | | |
| **As far as can be ascertained from the paper, how well was the study conducted? (see guidance notes)** | ++  +  − | Comments: Overall, the paper appears to be of high quality. |

Appendices

Appendix C: Example Data Extraction Form

Appendix D: PROSPERO Registration Summary

Appendix E: Research Ethics Declaration Form

Appendix F: Risk Assessment Form

Appendix G: Summary of Data Set Themes

Appendix H: Studies’ Reporting of PPIE

Appendix I: Report of Researchers’ Positionality & Reflexivity Statements

Appendix J: Sampling Strategy & Participant Selection Rationales

Appendix K: Risk of Bias Assessment Tool for Systematic Reviews (ROBIS)

\
